# Enantioselective Esterification of Ibuprofen under Microwave Irradiation

**DOI:** 10.3390/molecules18055472

**Published:** 2013-05-13

**Authors:** Zecheng Yang, Xuedun Niu, Xuedong Fang, Ge Chen, Hong Zhang, Hong Yue, Lei Wang, Dantong Zhao, Zhi Wang

**Affiliations:** 1 The Second Hospital of Jilin University, Changchun 130011, China; E-Mails: yangzechengne@163.com (Z.Y.); fangxuedong@medmail.com.cn (X.F.); 2 Key Laboratory of Molecular Enzymology and Engineering of Ministry of Education, College of Life Sciences, Jilin University, Changchun 130023, China; E-Mails: niuxd09@mails.jlu.edu.cn (X.N.); chenge0114@hotmail.com (G.C.); Zhanghong163163@163.com (H.Z.); xinqingtianlan@sina.cn (H.Y.); 3 Liaoning Key Laboratory of Urban Integrated Pest Management and Ecological Security, Shenyang University, Shenyang 110044, China

**Keywords:** ibuprofen, APE 1547, enzyme activity, microwave, enantioselectivity

## Abstract

Enantioselective esterification of ibuprofen has been successfully carried out in an organic solvent catalyzed by recombinant APE 1547 (a thermophilic esterase from the archaeon *Aeropyrum pernix* K1). Here we used microwave irradiation (MW) as the mode of heating to improve the enzyme performance. Under the optimum conditions, the enzyme activity of APE 1547 was 4.16 μmol/mg/h and the enantioselectivity (*E* value) was 52.9. Compared with conventional heating, the enzyme activity and the enantioselectivity were increased about 21.9-fold and 1.4-fold, respectively. The results also indicated that APE 1547 can maintain 95% of its activity even after being used five times, suggesting that the enzyme is stable under low power MW conditions.

## 1. Introduction

Many enzymes can now be used as biocatalysts for the synthesis of industrially important products in non-aqueous reaction media [1,2,3]. However, the bottleneck of non-aqueous enzymology is that enzymes usually exhibit poor activity [4], especially when the substrate is not their natural one. Therefore it is necessary to find a suitable method to accelerate the reactions and shorten the reaction times [5,6]. Over the years, many techniques have been developed to solve this problem, including lyophilization in the presence of some special additives [7,8] or the use of directed evolution to obtain more functional mutant enzymes [9]. Immobilization has also been used to improve the enzyme performance [10]. However, most techniques have yielded only modest improvements in enzyme activity and are generally not sufficient for large-scale and widespread application of non-aqueous bioprocessing.

Microwave irradiation (MW) has been proved to be a clean, fast, and convenient energy source [11,12] and is widely used in organic chemistry [13,14]. It’s generally believed that microwave irradiation can produce efficient internal heating by direct coupling of microwave energy with the molecules (solvents, reagents, catalysts) in the reaction mixture [15] and thus accelerate the reactions [16]. Since lipase-catalyzed reactions are rather sluggish in non-aqueous media, the microwave-assisted lipase-catalyzed reaction has developed quickly in the past few years. Yadav *et al.* [17] have reported that the initial activity for transesterification of methyl acetoacetate with various alcohols in the presence of immobilized lipases is increased in the 2.2–4.6 fold range by using microwave irradiation. Yu *et al*. [18,19,20] have also taken the advantage of microwave irradiation to enhance both the activity and enantioselectivity of Novozym 435 in the resolution of (*R,S*)-2-octanol in organic solvents and ionic liquids, respectively. However, there still remains relatively little research so far on the applications of microwave irradiation in lipase catalytic resolution reactions, and demonstration of their utility remains a pressing concern.

Ibuprofen is a widely used nonsteroidal anti-inflammatory drug with an asymmetric carbon in the C2 position. In our previous study, enantioselective esterification of ibuprofen has been successfully carried out in organic solvents using APE 1547 (a thermophilic esterase expressed in a recombinant *E. coli* BL21 harboring the APE1547 gene from the archaeon *Aeropyrum* *pernix* K1) and the reaction conditions for the acylation have also been optimized [21]. However, the activity was not satisfactory, even under the optimum conditions. In this study, we describe a temperature-controllable microwave assisted resolution of ibuprofen with APE1547 as the catalyst. A comparison between conventional heating and microwave irradiation on the resolution of ibuprofen has been performed and the reusability of APE1547 under MW has also been investigated.

## 2. Results and Discussion

### 2.1. Microwave Irradiation *versus* Conventional Heating

The enzymatic resolution of ibuprofen when heated by microwave irradiation and conventional heating was compared. As shown in Table 1, the microwave assistance was found to enhance both the enzyme activity and enantioselectivity, compared with the results obtained from the conventional heating. It was known that microwave heating involved directed absorption of energy by functional groups that bear ionic conductivity or a dipole rotational effect, and this energy was then released into the surrounding solution [15]. In our systems, the absorption of microwave irradiation caused the functional groups of the substrates to be of much higher reactivity than that incubated at the same temperature [22], and then the reaction rate was improved. One possible explanation for the enantioselectivity improvement was that MW caused a conformational change of APE 1547 which enhanced its enantioselectivity. The spectroscopy experiments were underway to clarify the mechanism behind these observed advantages.

**Table 1:** Comparison of the enzymatic resolution of ibuprofen heated by microwave irradiation and conventional heating *^a^*.

Heating method	Enzyme activity (μmol/mg/h)	Enantioselectivity (*E* value)
Conventional heating	0.19 ± 0.05	37.1 ± 1.6
Microwave irradiation	0.87 ± 0.04	47.5 ± 2.1

*^a^* The reactions were carried out in *n*-heptane (60 mL), ibuprofen (0.02 M) and 1-octanol (0.02 M) and APE 1547 (60 mg, pH 8.5). The mixture was incubated in a thermo-constant orbital shaker (60 °C, 150 rpm) or in the microwave oven (400 W, 60 °C, 150 rpm), respectively.

### 2.2. Effect of Microwave Power

**Figure 1:**
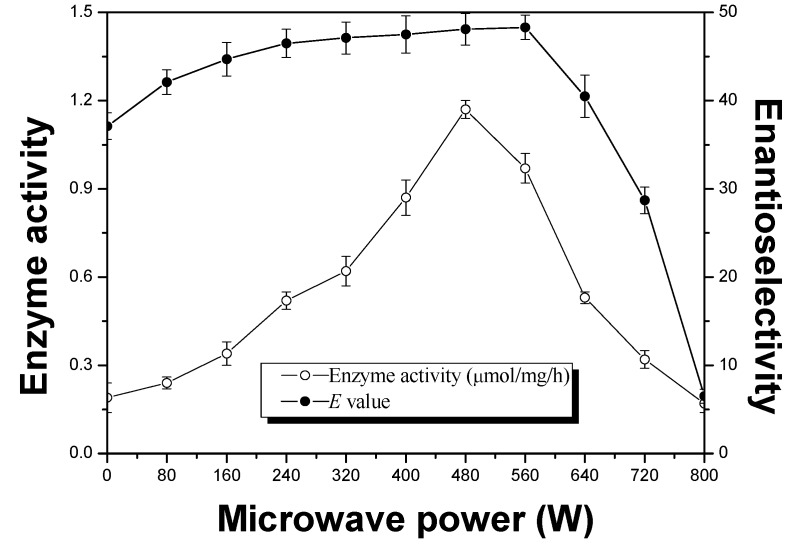
Effects of microwave power on enzyme activity (μmol/mg/h, ○) and enantioselectivity (*E* value, ●) in the enzyme-catalyzed esterification of ibuprofen. The reactions were carried out in *n*-heptane (60 mL), ibuprofen (0.02 M) and 1-octanol (0.02 M) and APE 1547 (60 mg, pH 8.5). The mixture was incubated in the microwave oven (60 °C, 150 rpm) with different microwave power.

To obtain the best performance of APE 1547 with the minimum energy consumption possible, the effect of microwave power in the range of 0–800 W was investigated. As shown in Figure 1, the enzyme activities exhibited a bell-shaped curve with changing the microwave power. When the microwave power was 480 W, the APE 1547 exhibited its highest activity. With a further increase or decrease in microwave power, the enzyme activity decreased. Generally, MW induces molecular rotation arising from dipole alignment with the external, oscillating electric field [23]. As a result, microwaves significantly impacted molecules with high dipole moments. All of the enzyme and substrate used in the present study had significant dipole moments. Thus the higher the microwave power set, the faster the dipole reorientated under the MW, which might make the functional groups obtain much higher reactivity, and gradually activate enzyme and substrate. Besides, no obviously change of enantioselectivity was found under low microwave power (<560 W). However, the *E* value decreased dramatically when the microwave power further increased. At high microwave power, the enzyme activity and the enantioselectivity decreased possibly due to denaturation of the enzyme caused by the quick change of temperature at the very beginning of the reaction. Based on the experimental results, 480 W was chosen as the optimum microwave power for this reaction.

### 2.3. Effect of Temperature

**Figure 2:**
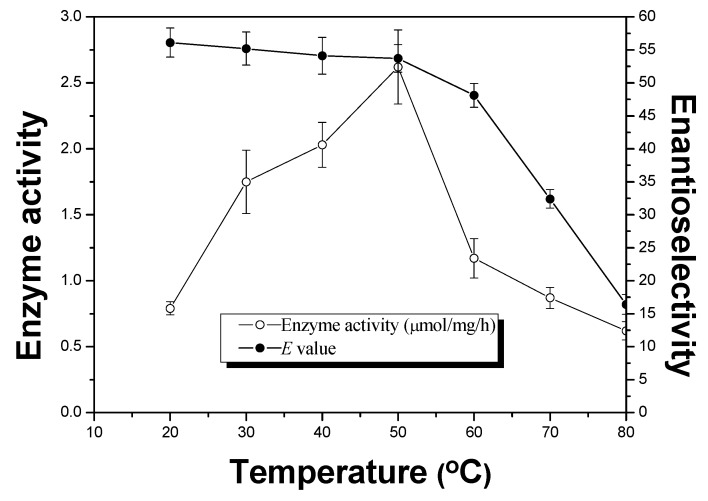
Effects of temperature on enzyme activity (μmol/mg/h, ○) and enantioselectivity (*E* value, ●) in the enzyme-catalyzed esterification of ibuprofen. The reactions were carried out in *n*-heptane (60 mL), ibuprofen (0.02 M) and 1-octanol (0.02 M) and APE 1547 (60 mg, pH 8.5). The mixture was incubated in the microwave oven (480W, 150 rpm) at different temperature.

It’s well known that most of the enzymes are readily deactivated by the fast enhancement of the temperature under microwave irradiation [24]. The problem can be solved by using immobilized enzyme which might exhibit high thermal stability than the free one [25,26]. Using thermophilic enzyme can also solve the problem. In this study, APE1547 as a thermophilic enzyme [27,28] was selected as the biocatalyst. The effect of temperature on the activity and enantioselectivity of APE 1547 under MW was examined in the range of 20–80 °C. As shown in Figure 2, the enzyme activity of APE1547 increased with the increase of temperature and the highest enzyme activity was observed at 50 °C. The possible explanation might be the thermal activation of APE1547 due to its thermophilic nature (its optimum temperature is about 90 °C) [27]. Another explanation could be attributed to the better solubility and diffusion of ibuprofen at higher temperatures in the reaction medium. When considering the enantioselectivity, high *E* value was observed at low temperatures, which has been well explained by Phillips [29]. Since the enzyme activity was observed to be greatest at 50 °C while maintaining a higher enantioselectivity, 50 °C was selected as the optimal temperature for this reaction.

### 2.4. Effect of pH

**Figure 3:**
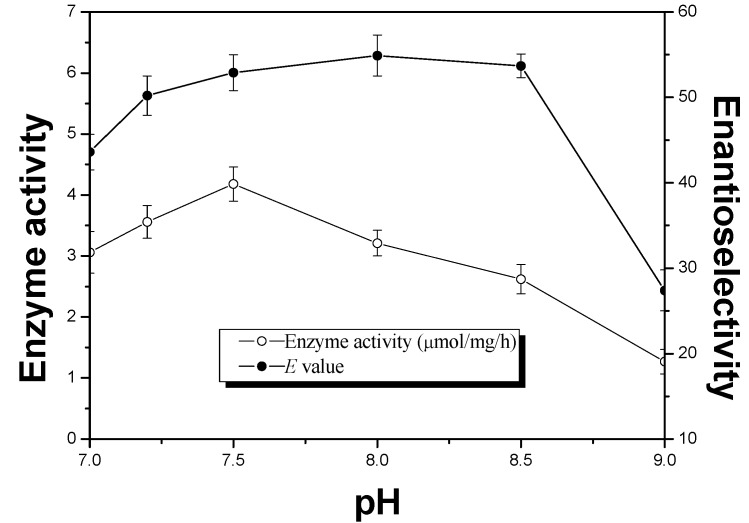
Effects of pH on enzyme activity (μmol/mg/h, ○) and enantioselectivity (*E* value, ●) in the enzyme-catalyzed esterification of ibuprofen. The reactions were carried out in *n*-heptane (60 mL), ibuprofen (0.02 M), 1-octanol (0.02 M), APE 1547 (60 mg) with different pH. The mixture was incubated in the microwave oven (480W, 50 °C, 150 rpm).

The pH of the aqueous solution may influence the ionization state of the residues of enzyme’s active-site. When the enzyme was lyophilized at its optimum pH and suspended in organic solvents, it could maintain its optimum ionization state and exhibit its best catalytic performance. This phenomenon is known as “pH memory” [30,31]. Thus, the enzyme performance can be improved by lyophilizing enzymes from aqueous solutions before using it in organic solvents. Here, we investigated the effect of the buffer pH used for preparation of the enzyme (Figure 3). It could be observed that the activity and the enantioselectivity were remarkably influenced by the pH of the aqueous solution from which the enzyme was prepared. The highest activity of the lipase was observed at pH 7.5, while the lipase showed maximum enantioselectivity at pH 8.0.

### 2.5. Effect of Enzyme Dosage

**Figure 4:**
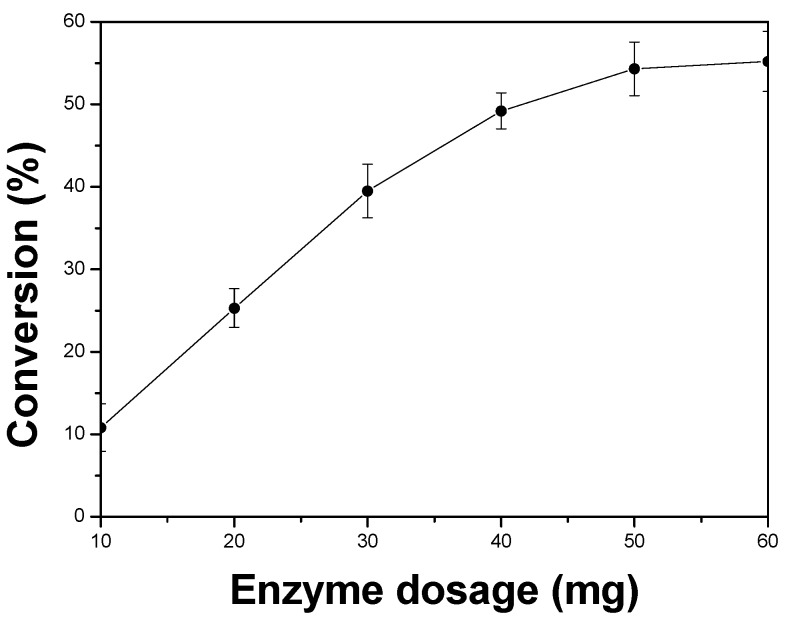
Effects of enzyme dosage on the enzyme-catalyzed esterification of ibuprofen. The reactions were carried out in *n*-heptane (60 mL), ibuprofen (0.02 M), 1-octanol (0.02 M) and APE 1547 (pH 7.5) with different enzyme dosage. The mixture was incubated in the microwave oven (480 W, 50 °C, 150 rpm) for 5.0 h.

In this study, the effect of enzyme dosage on the enantioselective esterification of ibuprofen was studied under microwave irradiation (Figure 4). As a result, the conversion rate increased gradually with increasing the enzyme dosage and 50 mg of the enzyme was sufficient for this reaction.

### 2.6. Reusability of Enzyme

The reusability of enzyme was carried out by using the recovered enzyme over several reaction cycles under microwave irradiation. As shown in Table 2, the enantioselectivity nearly kept constant and only slightly decrease (5%) was observed in enzyme activity under microwave irradiation after five reaction cycles. This indicates the possibility of reusing the enzyme under low power microwave irradiation, but it is still not ideal because the activity and enantioselectivity of the enzyme eventually decrease with successive reuse, probably due to the conformational changes. In order to improve the enzyme performance further, a study adopting the technique of immobilization is currently in progress and its results will be reported in due course.

**Table 2:** Reusability of APE 1547 under microwave irradiation ^a^.

Reaction Cycle	Relative activity (%)	*E* value
1	100	52.9 ± 1.6
2	98.9	52.3 ± 2.1
3	97.5	50.6 ± 2.4
4	96.4	51.8 ± 1.4
5	95.1	52.1 ± 2.5

*^a^* The reactions were carried out in *n*-heptane (60 mL), ibuprofen (0.02 M), 1-octanol (0.02 M) and APE 1547 (pH 7.5, 50 mg). The mixture was incubated in the microwave oven (480 W, 50 °C, 150 rpm) for 5.0 h. After each reaction cycle, the enzyme was recycled and repeatedly used in the next reaction.

## 3. Experimental

### 3.1. Catalysts and Chemicals

(*R*)-Fenoprofen, (*R,S*)-ibuprofen, (*S*)-(−)-1-(1-naphthyl)ethylamine [(*S*)-NEA] of analytical grade was purchased from Sigma-Aldrich-Fluka Chemical Co. (St. Louis, MO, USA). Other reagents of reagent grade were purchased from Shanghai Chemical Reagent Company (Shanghai, China). Solutions of triethylamine (TEA, 50 mM) and ethanolamine (EOA, 0.02 M) were prepared in acetonitrile. The solution of (*S*)-NEA (0.02 M) was prepared in acetonitrile-TEA (8:2, v/v). APE 1547 was home expressed from a newly constructed *E. coli* BL21 [27].

### 3.2. Microwave Equipment

Reactions were carried out in a commercial multimode microwave reactor (MCR-3, Shanghai JieSi Microwave Chemistry Corporation, Shanghai, China). The machine consisted of a continuous focused microwave power delivery system and an operator selectable power output from 0 to 800 W. The temperature of the reaction mixture was monitored and kept constant (±1 °C) by using a contact Teflon platinum resistance temperature transducer inserted directly into the reaction mixture. The content of the vessel was stirred by a rotating magnetic plate located below the floor of the microwave cavity and a Teflon-coated magnetic stir bar in the vessel.

### 3.3. Preparation of the Recombinant Enzyme APE1547

The recombinant *E. coli* BL21 harboring APE1547 gene from the archaeon *Aeropyrum* *pernix* K1 was constructed by Dr. R.J. Gao in our lab [27].The cells were cultured at 37 °C until OD_600_ reached 0.6. After IPTG was added to a final concentration of 1.0 mM, cultures were further incubated for 4 h. Cells were harvested by centrifugation, washed and resuspended in 50 mM Tris-HCl buffer (pH 8.5). The resuspended cells were disrupted by sonication and the homogenate was centrifuged at 12,000 rpm for 10 min to remove cell debris. The lysate was incubated at 37 °C for 30 min with 0.5 mg/mL of bovine DNase I, and then heated at 85 °C for 30 min. After being centrifuged at 12,000 rpm for 20 min, the supernatant was collected and lyophilized. The crude enzyme was partially purified by ammonium sulfate fractionation procedure and the ammonium sulfate precipitation was dialyzed in 50 mM Tris-HCl buffer (pH 8.5) to remove the excess salt. The enzyme was used after lyophilization.

To study the pH effect on synthesis, the enzyme powder (200 mg) was dissolved again in 50 mM Tris-HCl buffer (10 mL) with different pH values, and then lyophilized for 48 h to obtain the pH-adjusted enzyme.

### 3.4. Enzyme-Catalyzed Esterification of Ibuprofen

A standard reaction mixture consisted of *n*-heptane (60 mL), (*R,S*)-ibuprofen (0.02 M) and 1-octanol (0.02 M). The mixture was incubated in the microwave oven (480 W, 50 °C, 150 rpm). The reaction was started by the addition of APE1547 powder (50 mg, pH 7.5). All experiments were carried out in triplicate.

### 3.5. Reusability of Enzyme

After each batch, the lipase was recycled by centrifugation (10,000 rpm, 5 min, 4 °C) and washed with *n*-heptane for three times and dried in air at 20 °C. The recycled enzyme was pretreated to the desired pH. And then the enzyme was repeatedly used in the next reaction. The residue activity of the recycled enzyme was compared with the enzyme activity of the first cycle (100%).

### 3.6. Analytical Methods

The method of HPLC analysis was performed according to Canaparo *et al.* [32] with minor modifications. Periodically, the reaction mixture (50 μL) was withdrawn and mixed with the solution of (*S*)-NEA (100 μL). After standing for 3 min, the solution of EOA (100 μL) was added to stop the reaction further. The solution was transferred into an amber glass vial for automatic injection into the HPLC system.

The reaction mixture analysis was performed on an Agilent 1200 HPLC with a YMC C18 column (150 mm × 4.6 mm; Greenherbs Co. Ltd, Beijing, China) at a flow rate of 1.6 mL/min. The mobile phase was a mixture of acetonitrile–water–acetic acid–triethylamine (60:40:0.1:0.02, v/v/v/v; pH 5.0). UV detection at 280 nm was used for quantification at ambient temperature and (*R*)-fenoprofen was used as internal standard (its retention time is about 9.8 min). The injection of samples (20 μL) was performed using an autosampler (Jasco 851-AS, Tokyo, Japan). Before the next sample injection, the system must be pre-equilibrated with the starting mobile phase for at least 5 min. The retention time of the (*S*)-ibuprofen and (*R*)-ibuprofen was 15.1 min and 16.8 min, respectively.

The enantiomeric excess of the remained (*S*)-ibuprofen (*ee*) was determined by calculating the peak areas of the enantiomers, and the conversion (*C*) were determined based on the decrease of ibuprofen. The enzyme activity (μmol/mg/h) was defined as the amount (in micromoles) of ibuprofen ester produced per hour per milligram of enzyme. The enantiomeric ratio (*E* value) was calculated according to Chen *et al.* [33]:

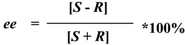


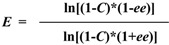

where *S* and *R* represent the concentrations of the (*S*)-enantiomer and (*R*)-enantiomer, respectively.

## 4. Conclusions

In this study, we used microwave irradiation (MW) to improve the enzyme activity and enantioselectivity of APE 1547 in the enzymatic resolution of ibuprofen. The optimum conditions involved the use of low power microwave (480 W) at 50 °C. Furthermore, 50 mg APE 1547 (pH 7.5) was sufficient for this reaction system. Compared with conventional heating, the enzyme activity and the enantioselectivity increase 21.9-fold and 1.4-fold, respectively. The results also indicated that the enzyme is stable under low power MW conditions and could be reused.
